# The effectiveness of moxibustion for treating of low back pain: A protocol for systematic review and meta-analysis

**DOI:** 10.1097/MD.0000000000022522

**Published:** 2020-10-23

**Authors:** Siyuan Zhu, Jun Xiong, Jun Chen, Genhua Tang, Zhiying Zhong, LunBin Lu, Xingchen Zhou, Han Guo, Hao Fan

**Affiliations:** a Jiangxi University of Traditional Chinese Medicine; b The Affiliated Hospital of Jiangxi University of Traditional Chinese Medicine, Nanchang, China.

**Keywords:** low back pain, meta-analysis, moxibustion, myofascitis, protocol, systematic review

## Abstract

**Background:**

Low back pain is a common clinical chronic disease with symptoms of back soreness, numbness, and pain. The incidence of low back pain is high, and gradually increases with age. It is mainly middle-aged and has a high recurrence rate. It is considered to be one of the common diseases with the highest disability rate. The aim of this systematic review is to assess the effectiveness and safety of moxibustion therapy for low back pain.

**Methods:**

Two reviewers will electronically search the following databases: the Cochrane Central Register of Controlled Trials (CENTRAL);PubMed; EMBASE; China National Knowledge Infrastructure (CNKI); Chinese Biomedical Literature Database (CBM); Chinese Scientific Journal Database (VIP database); and Wan–Fang Database from the inception, without restriction of publication status and languages. Additional searching including researches in progress, the reference lists and the citation lists of identified publications. Study selection, data extraction, and assessment of study quality will be performed independently by 2 reviewers. If it is appropriate for a meta-analysis, RevMan 5.4 statistical software will be used; otherwise, a descriptive analysis will be conducted. Data will be synthesized by either the fixed-effects or random-effects model according to a heterogeneity test. The results will be presented as risk ratio (RR) with 95% confidence intervals (CIs) for dichotomous data and weight mean difference (WMD) or standard mean difference (SMD) 95% CIs for continuous data.

**Results:**

This study will provide a comprehensive review of the available evidence for the treatment of moxibustion with low back pain.

**Conclusions:**

The conclusions of our study will provide an evidence to judge whether moxibustion is an effective and safe intervention for patients with low back pain.

**Trial registration number:**

INPLASY202080027.

## 1 Introduction

### 1.1 Description of the condition

Low back pain refers to the long-term chronic strain of lumbar muscles, myofascial membranes, tendons, and cutaneous nerves, leading to aseptic inflammation, tissue congestion, edema, adhesion, fibrosis, and long-term development of cord-like nodules.^[1,2]^ The main manifestation of this disease is low back pain (LBP), accompanied by dysfunction and autonomic dysfunction,^[3,4]^ which is the main cause of non-specific low back pain(NLBP). Low back pain is a common cause of restricted activities and affecting life. It is the most common musculoskeletal disease, with an incidence of up to 70% in adults. As of 2016, the global prevalence and incidence of low back pain reached 51104.8 and 250,277,000 respectively.^[5]^ In addition, in most parts of the world, low back pain is the main reason that restricts peoples activities, lacks work, and reduces productivity.^[6]^ The incidence of low back pain is high, and gradually increases with age. It is mainly middle-aged and has a high recurrence rate. It is considered to be one of the common diseases with the highest disability rate. Have a great impact on their daily work and life.^[7]^ The purpose of intervention for low back pain is to relieve pain and improve the state of restricted functional activity. Usually, the first choice is drug therapy and non-invasive treatment.^[8]^ The most commonly used to relieve pain syndrome are non-steroidal anti-inflammatory drugs (NSAIDs), but their long-term use may have side effects on the gastrointestinal tract, kidneys, and antiplatelets. Therefore, doctors and patients should be cautious in clinical treatment. ^[9,10]^ Moxibustion, as a commonly used treatment method within the scope of traditional Chinese Medicine, has a good effect on the treatment of various types of pain diseases, and has been widely accepted and recognized worldwide.^[11]^

### 1.2 Description of the intervention

This disease belongs to the categories of low back pain, arthralgia and so on in Chinese medicine. The internal causes are mostly due to liver and kidney deficiency, weak qi and blood, trauma or strain. The external causes are mostly feelings of wind, cold, dampness, and trauma, which lead to poor circulation of qi and blood, pain caused by impassability, blocked veins, and muscle pain after a long period of time.^[3]^ Moxibustion therapy is an external treatment method, which is mainly made from a series of Chinese herbal medicines such as mugwort leaves. It uses moxibustion to generate warmth on the patients local skin, and then exerts a therapeutic effect.^[12,13]^ The basic theory of traditional Chinese medicine believes that moxibustion has the effects of warming the meridians and dispelling cold, dispelling dampness and relieving pain. It can play an important role in preventing and treating diseases.^[14]^ So far, many systematic reviews have shown that moxibustion therapy in traditional Chinese medicine has a good effect on the treatment of painful diseases.^[15,16]^ Our research group has repeatedly checked the literature and found that moxibustion has a certain effect on the treatment of low back pain in terms of clinical efficacy and alleviating the development of pain. Therefore, moxibustion may become an effective treatment to delay the progression of low back pain.

### 1.3 How the intervention might work

In traditional Chinese medicine, moxibustion is considered to play a major role through warming and drug effects. In Western medicine, the mechanism of moxibustion is not clear.^[17]^ Modern medical research has confirmed that moxibustion has anti-inflammatory, expelling rheumatism, dredging collaterals and relieving pain, improving the body's immunity, and achieving the effect of treating both symptoms and root causes.^[18–22]^

### 1.4 Why it is important to do this review

Nonsteroidal anti-inflammatory drugs (NSAIDs) have been shown to be very effective in the treatment of low back pain, but their side effects and adverse reactions are still a major problem for some patients.at the same time.A systematic evaluation and meta-analysis showed that acupuncture treatment myofascitis may be effective, ^[23]^ but there is a large difference between acupuncture and moxibustion, which requires additional evaluation. Therefore, we hope to conduct a systematic evaluation of the published literature, with the aim of exploring the efficacy and safety of moxibustion in the treatment of low back pain, and to draw more reliable conclusions through meta-analysis to provide concluding evidence for clinical practice.

### 1.5 Objectives

To systematically evaluate the effectiveness and safety of moxibustion therapy for low back pain patients.

## 2 Methods and analysis

This protocol was designed in accordance with the methodological guidelines for overviews provided by the Cochrane Handbook for Systematic Reviews of Interventions.^[24]^ It is registered on the International Prospective Register of Systematic Reviews. (Registration number INPLASY202080027; https://inplasy.com/inplasy-2020–8-0027/).

### 2.1 Inclusion criteria for study selection

#### 2.1.1 Types of studies

Randomized controlled trials (RCTs) will be included, without restrictions on publication status.

#### 2.1.2 Types of participants

Adult patients with low back pain, regardless of sex, race, or educational and economic status.

#### 2.1.3 Types of interventions

Research using moxibustion or combined with other therapies, without limiting the treatment time and dose.

#### 2.1.4 Types of comparisons

The control group's treatment is not limited, including no treatment, placebo, or any control considered for comparison in a single systematic review.

#### 2.1.5 Types of outcome measures

Primary outcomes: Visual Analogue Scale (VAS score).

Secondary outcomes:

1. Dysfunction index score (ODI score).

## 3 Clinical efficacy

### 3.1 Search methods for identification of studies

#### 3.1.1 Electronic searches

The following databases from the inception to July 2020 will be searched by 2 independent reviewers, without restriction to publication status and languages: the Cochrane Central Register of Controlled Trials (CENTRAL); PubMed; EMBASE; China National Knowledge Infrastructure (CNKI); Chinese Biomedical Literature Database (CBM); Chinese Scientific Journal Database (VIP database); and Wan–Fang Database. A search strategy for PunMed database, which is established according to the Cochrane handbook guidelines, is shown in Table 1. Similar search strategies will be applied for the other databases. Before this review completed, the 2 reviewers will conduct the searching once again to ensure the latest studies could be included.

**Table 1:**
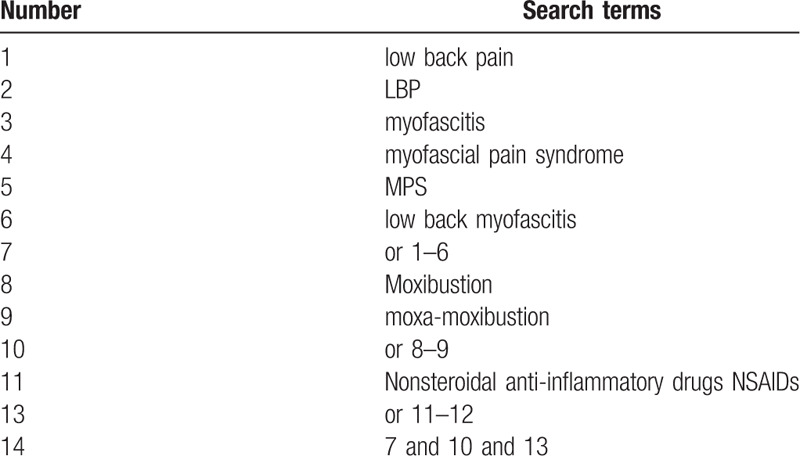
Search strategy for the PubMed database.

#### 3.1.2 Searching other resources

Besides, electronic sources for relevant researches in progress will also be searched, including Clinicaltrials.gov (http://www.clinicaltrials.gov) and the World Health Organization International clinical trials registry search portal (http://apps.who.int/trialsearch/). Additionally, the citation list will be retrieved in Web of Science. Besides, the reference lists of those studies meeting the inclusion criteria and relevant systematic reviews will also be identified for additional relevant studies.

### 3.2 Data collection and analysis

#### 3.2.1 Selection of studies

**Figure 1:**
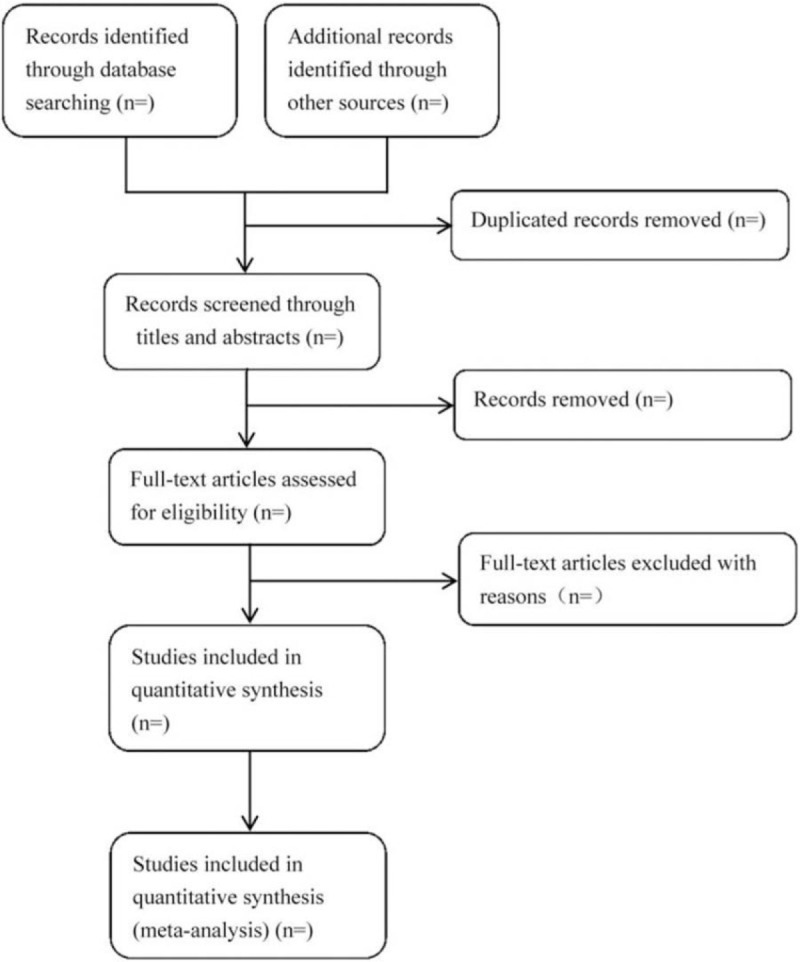
Flowchart of literature selection.

We plan to conduct this systematic review between October 30, 2020 and July 30, 2022. All reviewers have undergone a training to ensure a basic understanding of the background and purpose of the review. After electronic searching, the records will be uploaded to a database set up by EndNote software (V.X7). Records selected from other sources will also be moved to the same database. Two reviewers (SYZ and GHT) will independently screen the titles, abstracts, and keywords of all retrieved studies and decide which trials meet the inclusion criteria. We will obtain the full text of all possibly relevant studies for further assessment. Excluded studies will be recorded with explanations. Any disagreements will be resolved by discussion between the 2 reviewers (SYZ and GHT) and the third author (JC) for arbitration when necessary. We will contact reviewers of trials for clarification when necessary. The study flow diagram is shown in Fig. 1.

### 3.3 Data extraction and management

A unified data extraction form will be designed by all of the reviewers and its applicability will be tested in a small scope of trials by 2 reviewers (ZYZ and LBL). They will then independently extract data in the following domains: general information, participants, methods, interventions, outcomes, results and other information. Any disagreement will be discussed between the 2 reviewers, and further disagreements will be arbitrated by the third author (JC).

### 3.4 Assessment of risk of bias in included studies

The risk of bias will be assessed by 2 reviewers (XCZ and HG) with the Cochrane Collaborations tool for risk of bias assessment. The risk of bias in included studies will be evaluated according to the following aspects: sequence generation, allocation sequence concealment, blinding of participants and personnel and outcome assessors, incomplete outcome data, selective outcome reporting, and other sources of bias. The assessments will be classified into 3 levels: low risk, high risk, and unclear risk.

### 3.5 Measures of treatment effect

RevMan V.5.4 will be used for data analysis and quantitative data synthesis. For continuous data, we will use standard mean difference (SMD) to measure the treatment effect with 95% confidence intervals (CIs). For dichotomous data, a risk ratio (RR) with 95% CIs for analysis will be adopted.

### 3.6 Unit of analysis issues

Data from studies with parallel-group will be included for meta-analysis. For randomized cross-over trials, only the first phase data will be included. In these trials, participants are individually randomized to 2 intervention groups, and a single measurement of each outcome from each participant is collected and analyzed.

### 3.7 Dealing with missing data

We will try to contact the first or corresponding authors of the included studies by telephone or email to retrieve missing or insufficient trial data. If missing data are unavailable, we will make an assumption using the terms “missing at random” and “not missing at random” to represent different scenarios, which is recommended in the Cochrane Handbook.^[25]^ For the data “missing at random”, only the available data will be analyzed. For the data “not missing at random”, we will displace the missing data with replacement values and a sensitivity analysis will be used to determine whether the results are inconsistent.

### 3.8 Assessment of heterogeneity

On the basis of the data analysis, random effect or fixed effect models will be employed according to the heterogeneity given by *I*^*2*^ statistic value. To be concrete, a fixed effect model will be adopted if the heterogeneity is indicated as high (*I*^*2*^ < 50%); otherwise, a random effect model will be applied on the contrary.

### 3.9 Assessment of reporting biases

We will use funnel plots to detect reporting biases and small study effects. If more than 10 studies are included in the meta-analysis, we will conduct a test for funnel plot asymmetry using Egger method. All eligible trials will be included, regardless of their methodological quality.

### 3.10 Data synthesis

The systematic review will be conducted with the use of RevMan 5.4. Taking account of the heterogeneity assessment, MD or RR with fixed or random effect model will be computed. Additionally, if heterogeneity is considered significant, the sensitivity or subgroup analysis will be generated to distinguish the source of it. When it comes to the situation that the data are insufficient for quantitative analysis, the review will only represent and summarize the evidence.

### 3.11 Sensitivity analysis

Sensitivity analysis will be conducted to validate the robustness of the primary results. We will exclude certain trials by a revaluation of methodological quality, study types, sample size, missing data or other possible factors. Careful interpretations will be employed for sensitivity analysis if differ substantially.

### 3.12 Grading the quality of evidence

The Grading of Recommendations Assessment, Development and Evaluation (GRADE) working group methodology will be applied for the quality of evidence for all outcomes.^[26]^ Six domains will be assessed, containing risk of bias, consistency, directness, precision, publication bias and additional points. The assessments will be categorized into 4 levels: high, moderate, low, or very low.

### 3.13 Subgroup analysis

If data are available, a subgroup analysis will be performed based on the type of moxibustion intervention (moxibustion with moxa stick, direct moxibustion, indirect moxibustion, etc) because this is the main factor causing heterogeneity.

## 4 Discussion

Low back pain is mainly due to the invasion of wind, cold and moisture, which makes the meridian blood in the muscles and muscles not smooth, and It is prone to long-term indoor office work, sedentary sitting, improper sitting and other people, causing chronic strain. Or the acute muscle injury caused by trauma is not effectively treated. The disease can cause pain in the patients muscle tissue, and long-term edema can cause local tissue ischemia and hypoxia, resulting in tissue fibrous adhesions.^[27–28]^Therefore, the key to treating this disease with Chinese medicine is to promote blood circulation, remove blood stasis and relieve pain. Acupuncture and moxibustion therapy can clear the blood, relieve nerve root compression, promote blood circulation in the waist, and eliminate edema by stimulating acupoints to relieve pain.^[28,29]^ Though nonsteroidal anti-inflammatory drugs (NSAIDs) have been recommended to be the commonly used for the management of low back pain, the side effects burden is still concerned.^[10]^ Therefore, it is very meaningful to explore the effectiveness and safety of moxibustion in the treatment of low back pain.

## Author contributions

**Conceptualization:** Siyuan Zhu, Jun Xiong.

**Data curation:** Siyuan Zhu, Jun Chen, Genhua Tang, Zhiying Zhong, LunBin Lu, Xingchen Zhou, Han Guo.

**Formal analysis:** Siyuan Zhu, Jun Chen.

**Investigation:** Jun Xiong, Genhua Tang, Zhiying Zhong, Hao Fan.

**Methodology:** Siyuan Zhu, Jun Chen, LunBin Lu.

**Software:** Siyuan Zhu, Jun Chen.

**Supervision:** Jun Xiong, LunBin Lu, Hao Fan.

**Writing – original draft:** Siyuan Zhu, Jun Xiong.

**Writing – review & editing:** Jun Xiong, Jun Chen, Genhua Tang, LunBin Lu, Hao Fan.
